# O-WCNN: an optimized integration of spatial and spectral feature map for arrhythmia classification

**DOI:** 10.1007/s40747-021-00371-4

**Published:** 2021-04-26

**Authors:** Manisha Jangra, Sanjeev Kumar Dhull, Krishna Kant Singh, Akansha Singh, Xiaochun Cheng

**Affiliations:** 1 Department of Electronics and Communication Engineering, Guru Jambheshwar University of Science and Technology, Hisar, Haryana India; 2 Faculty of Engineering and Technology, Jain (Deemed-to-be University), Bengaluru, India; 3 Department of Computer Science Engineering, ASET, Amity University Uttar Pradesh, Noida, India; 4 Department of Computer Science, Middlesex University, London, UK

**Keywords:** Deep learning, ECG, Arrhythmia, Depthwise separable convolution, CNN, Wavelet transform

## Abstract

The regular monitoring and accurate diagnosis of arrhythmia are critically important, leading to a reduction in mortality rate due to cardiovascular diseases (CVD) such as heart stroke or cardiac arrest. This paper proposes a novel convolutional neural network (CNN) model for arrhythmia classification. The proposed model offers the following improvements compared with traditional CNN models. Firstly, the multi-channel model can concatenate spectral and spatial feature maps. Secondly, the structural unit is composed of a depthwise separable convolution layer followed by activation and batch normalization layers. The structural unit offers effective utilization of network parameters. Also, the optimization of hyperparameters is done using Hyperopt library, based on Sequential Model-Based Global Optimization algorithm (SMBO). These improvements make the network more efficient and accurate for arrhythmia classification. The proposed model is evaluated using tenfold cross-validation following both subject-oriented inter-patient and class-oriented intra-patient evaluation protocols. Our model achieved 99.48% and 99.46% accuracy in VEB (ventricular ectopic beat) and SVEB (supraventricular ectopic beat) class classification, respectively. The model is compared with state-of-the-art models and has shown significant performance improvement.

## Introduction

Heart disease has been one of the leading causes of increased mortality in lower and middle-income countries. As per reports of the World Health Organization (WHO), approximately 17.9 million people die yearly due to cardiovascular diseases (CVD) in the world [1]. In 2020, CVD is the common comorbidity in COVID-19 increasing the risk of death by 12-fold [2]. Awareness about the risk factors and accurate CVD is the only way to reduce the mortality rate.

**Fig. 1:**
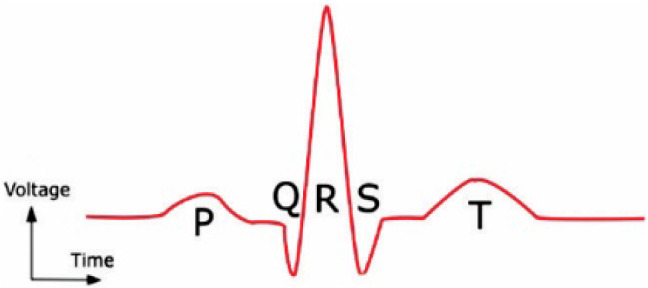
A standard ECG heartbeat composed of P wave, QRS complex, and T wave

Several invasive and non-invasive techniques such as the electrocardiogram (ECG) [3, 4], X-ray coronary angiography (XRA) [5], and Ultrasound imaging [6] are available to identify an anomaly in the electrical and mechanical operation of the heart. However, Electro-cardio-gram (ECG) is the primary, non-invasive, and inexpensive technique popular for heart abnormality diagnosis. A typical ECG waveform is composed of P, QRS complex, and T wave as shown in Fig. 1. An electrocardiogram is a graphic recording of electrical impulses and stimulus variations to the time captured using electrodes. Any variation in the source, rhythm, and rate of these electrical stimuli is reflected in the characteristic P-QRS-T waveforms that imply cardiac arrhythmia [7]. The dominant existence of chronic arrhythmic beats like ventricular fibrillation (VF) and premature ventricular contraction (PVC) are indicators of chronic CVD like cardiac arrest. Therefore, regular monitoring of cardiac arrhythmia is necessary for reducing the mortality rate due to CVD.

The biomedical research community has developed various automatic ECG arrhythmia classifiers based on pattern recognition in the last 50 years [8]. The conventional classifiers were variants of neural networks, and other diagnosis statistical pattern recognition methods such as support vector machines (SVM) [9], random forest algorithms [10], feed-forward neural network (FFNN) [11, 12], radial basis function neural network (RBFNN) [13], probabilistic neural network (PNN)[14] and many others. The conventional classifiers’ performance is supplemented using feature extraction and feature selection techniques. The popular feature extraction and selection techniques include empirical mode decomposition (EMD) [15], wavelet decomposition [12], Hjorth parameters [16], discrete cosine transform (DCT) [9], higher-order statistics (HOS) [17], principal component analysis (PCA) [9, 12], ant colony optimization (ACO) [18], particle swarm optimization (PSO) [19].

Since the last decade, the development in deep learning has oriented research towards the designing of modern classifiers. The modern classifiers are based on deep neural networks and can automatically extract the feature map. Therefore, the need for an efficient feature extraction technique is eliminated in modern classifiers. Convolutional neural networks (CNN) [20–22], multi-scale and multi-channel convolutional neural network (MSCNN, MWCNN, and DeepArrnet) [23–25], long short term memory (LSTM) [26] stacked denoising autoencoder [27], recurrent neural network (RNN) [28] are some of the modern classifiers available in the literature. A two-stage hierarchical deep CNN model is proposed in [29] to classify ECG signals into 16 arrhythmia classes. The fundamental building block of modern classifiers is the convolution layer. Mostly convolutional neural networks use the Conv1D (1-dimensional convolution) layer. However, inspired by the performance of 2-D CNN in computer vision applications, models using Conv2D layers are explored for the ECG arrhythmia classification problem [30, 31]. The image of ECG signals is used as input in [30]. Whereas [31] proposed converting 1-D ECG signal into 2-D dual heartbeat coupling matrix to use as input in 2-D CNN.

The complex and deep models based on conventional convolution layer Conv1D are computationally expensive and lead to overfitting data [32, 33]. Thus, it reduces the generalization capability of the network. However, the invention of the Xception network and the concept of depth-wise separable convolution layers resolve the problem by reducing the computational burden and offers performance enhancement by efficient utilization of parameters as experimented on ImageNet and JFT dataset [34]. Inspired by the performance of the Xception network, Mahmud et al. proposed deep CNN architecture called DeepArrNet using depthwise separable convolution layers [25]. The promising performance of DeepArrNet is reported in the literature with a shallow network of only 238,629 parameters. Similarly, a network equipped with PDblocks called PDNet is proposed in [35]. A PDblock is a cascade of pointwise convolution and depthwise convolution layers.

Most models are trained on ECG morphological segments directly and learn only spatial features using stacked convolution layers. However, such methods have shown higher sensitivity for either VEB or SVEB class [20]. Therefore, concatenation of spectral features and spatial features using a multi-scale convolutional network is proposed in [24].

The decade of research in deep learning has resulted in plenty of architectures with outstanding performance. But there are chances of over-estimation of performance of existing models available in literature by testing on carefully selected data. The class-oriented evaluation protocol selection also results in an over-estimation of performance using the same patient’s data in the training and test set, which is significantly less probable in practical systems. Some of the high-performing models are implemented using active learning and patient-specific approaches, which are useful but computationally expensive due to the need for real-time expert annotations of misclassified beats repeatedly for training the system. Therefore, there is still room for efficient and optimized networks equally and highly sensitive to all types of arrhythmic beat classification implemented using an inter-patient evaluation approach.

This paper proposes an efficient multi-channel convolutional neural network designed using depthwise separable convolution layers to learn from spatial and spectral feature maps. The proposed model, named ‘O-WCNN’ is designed using the methodology proposed in our prior work [36] to optimize the model’s depth and architecture. The basic unit of the proposed network is the depthwise separable convolution layer. The network hyperparameters are optimized using the ‘Hyperopt’ library [37]. The objective of the model is the classification of heartbeats into four categories named N (normal), VEB (ventricular ectopic beat), SVEB (supraventricular ectopic beat), and F (fusion beat) as per AAMI standard. The model performance is evaluated using both inter-patient protocol and class-oriented intra-patient protocol to compare with existing literature.

The article is organized into eight sections, including the introduction section. The methodology and workflow are described in “Methods”. The data preparation methods such as pre-processing and segmentation are discussed in “Data preparation”. The basic framework of model design is represented in “Proposed model”. The final model design and its hyperparameters are discussed in the subsequent section. “Experiment and evaluation” describes the experimental environment, dataset, performance metrics used for experimentation and evaluation. The results are discussed in “Results and discussion”. The conclusive remarks are given in the last section.

## Methods

**Fig. 2:**
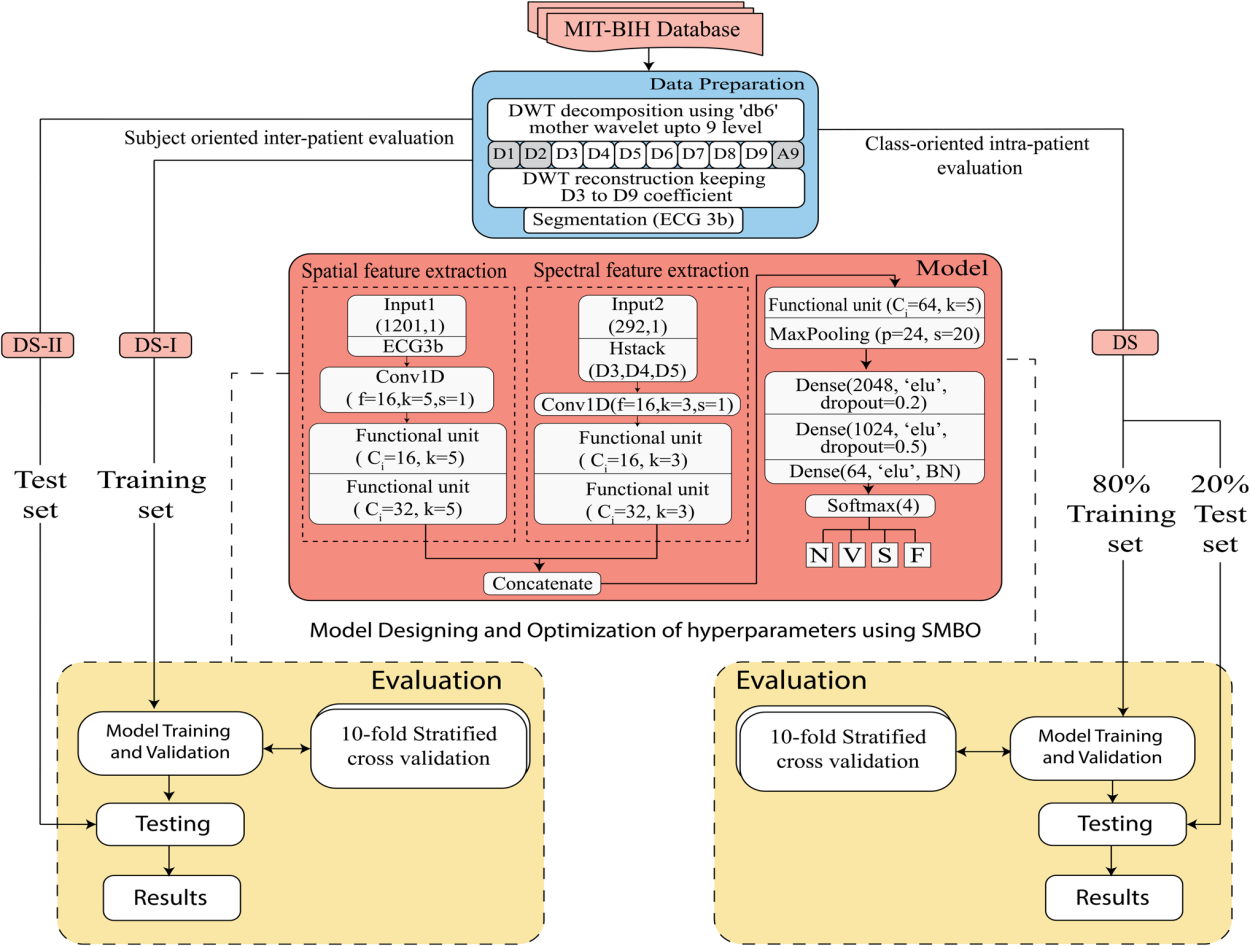
Workflow of proposed method

In this work, we have proposed an optimized model based on deep learning for the diagnosis of cardiac arrhythmia. The proposed model tends to learn from the spatial and spectral features and classify the signal into four arrhythmia types. The spatial features are extracted using depthwise separable convolution layers. The spectral features are extracted using the discrete wavelet transform (DWT) based signal decomposition detail coefficients D3–D5. The selective choice of coefficients is to extract spectral features from the QRS complex. The spectral and spatial features are concatenated and classified using a convolutional neural network. The workflow of the proposed method is shown in Fig. 2.

The methodology can be divided into the following phases: (1) data preparation, (2) Model design, and (3) Model evaluation.

In the first phase, the data from the MIT-BIH database is denoised using wavelet-based pre-processing. The clean recordings are prepared for experimentation using segmentation such that each segment has three ECG beats. The respective target label is stored in the target vector. Then based on the evaluation approach, the dataset is organized into the training set and test set.

In the second phase, the multi-channel model is designed and optimized using hyperparameter optimization. The segmented 3-beat ECG signal is given as input on one channel for spatial feature map extraction. The spectral feature map is generated using horizontally stacked wavelet transformed detail coefficients D3, D4, and D5 as input on another channel. Each functional unit comprises a cascade of two depth-wise separable layers, each followed by a batch normalization and activation layers. The hyperparameters of the model are optimized using the Sequential Model Based Global Optimization algorithm (SMBO).

In the third phase, the model is evaluated using two approaches: the subject-oriented inter-patient approach and the class-oriented intra-patient approach. The model training, validation, and testing have been done using tenfold stratified cross-validation. Each of the phases and related material is explained in the following sections.

## Data preparation

### Wavelet based denoising

Many MIT-BIH database recordings are corrupted with artifacts such as baseline wander, muscle artifacts, and power line interference (PLI). Baseline wander is a low-frequency noise causing signal drift above or below the zero-axis value. This drift affects the reading of fiducial points, mainly R peak and hence segmentation also. Further, PLI appears as high-frequency impulses lying in the 50–60 Hz range in ECG signal caused by the coupling of human body distribution capacitance with power lines connected with the ECG recording instrument [3].

For reducing the effect of these noises, we have implemented a wavelet-based pre-processing technique as proposed in [38]. The signal is filtered using a multi-resolution wavelet analysis. The raw signal is decomposed up to nine levels of decomposition using ‘db6’ mother wavelet. The mother wavelet chosen has morphological similarity with the signal in interest. Baseline wander, and PLI is removed by nullifying the detail and approximation coefficients falling in the frequency range of artifacts and then reconstructing back the signal. The frequency range of all the detail and approximation coefficients is given in Table 1.

**Table 1:** Frequency range of wavelet decomposed signal (Fs = 360 Hz) [36]

Level	Detail coefficient frequency range (Hz)	Approximation coefficient frequency range (Hz)
1	90–180	0–90
2	45–90	0–45
3	22.5–45	0–22.5
4	11.25–22.5	0–11.25
5	5.625–11.25	0–5.625
6	2.81–5.625	0–2.81
7	1.4–2.8	0–1.4
8	0.7–1.4	0–0.7
9	0.35–0.7	0–0.35

### Segmentation

In this paper, 3-beat segments are extracted from 30 min long recordings of the database. We have adopted a segmentation technique proposed in [39]. The technique is implemented in two steps: (1) segment extraction and (2) segment alignment. First, variable-length segments are extracted based on the location of R-peaks. In this paper, 3-beat segments are extracted for each beat. Then, variable-length segments are converted to fixed-length segments using segment alignment.

For 3-beat *i*th segment extraction, the samples are taken in the range from.

$$\left\{{{{R}}}_{{{i}}}- \left\lfloor{\frac{1}{2}\left({{{R}}}_{{{i}}-1}-{{{R}}}_{{{i}}-2}\right)}\right\rfloor\right\} \text{ to } \left\{{{{R}}}_{{{i}}}+ \left\lfloor{\frac{1}{2}\left({{{R}}}_{{{i}}+2}-{{{R}}}_{{{i}}+1}\right)}\right\rfloor \right\}.$$

**Fig. 3:**
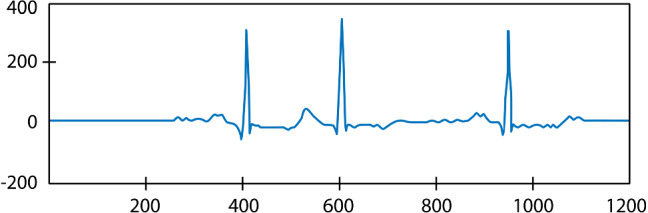
Three beat segment (ECG3b) segmented from recording number 100 of MIT-BIH database

Such R-peak dependent extraction results in segments of variable length. Such a technique ensures minimum loss of information due to heart rate variability (HRV) compared to fixed-length segmentation approaches [14]. However, the model accepts fixed-length signals. Therefore, in the alignment phase, the length of each three-beat segment is fixed to 1201 samples by keeping the ${{{R}}}_{{{i}}}$ peak at the center (sample 601). The signals are cropped/padded if they are longer/shorter than 1201 samples. The length of all segments is decided to be greater than the length of 95% of extracted segments [36]. Therefore, only less than 5% of the signals are cropped in the alignment phase. An example 3beat segment is shown in Fig. 3.

### Standardization

In this work, the training, validation, and test sets are standardized using the standard scaler method. The Test set has been standardized using statistics of the training dataset as the distribution of test signals is not known before-hand in the real-time testing scenario. Standardization increases convergence speed. The standard scaler method is mathematically represented by Eq. (1).

1 $$ \text{Standardized} (X) = \frac{{X - {\text{mean}}(X)}}{{{\text{std}}(X)}}. $$

## Proposed model

### Problem statement

This paper aims to design an optimized ECG arrhythmia classifier model using depthwise separable convolution layers known as the separable Conv1D layer. Further, the model design is such that it learns spatial and spectral feature maps from the ECG signal. Depthwise separable convolution layers offer efficient use of parameters in learning spatial features. The use of wavelet transformed detailed coefficient as input to the convolutional neural network allows learning of spectral features. The model tends to learn from the given training set $X$, here *X* is composed of morphological ECG signal vector and multi-resolution wavelet decomposition coefficients denoted as $[x_{m}^{(i)} ,x_{w}^{(i)} ]$, and class label $y^{(i)}$.

2 $$ X = \left\{ \begin{gathered} ([x_{m}^{(1)} ,x_{w}^{(1)} ],y^{(1)} ),([x_{m}^{(2)} ,x_{w}^{(2)} ],y^{(2)} ),... \\ ([x_{m}^{(k)} ,x_{w}^{(k)} ],y^{(k)} ) \\ \end{gathered} \right\}. $$

During the training phase, the model learns and updates its weight parameters iteratively. The objective is to minimize the cost function. Here, the cross-entropy function is used as the cost function, given by Eq. (3).

3 $$ L(X) = - \frac{1}{k}\sum\nolimits_{j = 1}^{m} {y^{(i)} \log (\overset{\lower0.5em\hbox{$\smash{\scriptscriptstyle\frown}$}}{y}^{(j)} )} . $$

Here *k* is the number of training instances,$$y^{(j)}$$ is target one hot encoded vector where $$j \in (1,m)$$ $$\overset{\lower0.5em\hbox{$\smash{\scriptscriptstyle\frown}$}}{y}^{(j)}$$ is estimated probability of belonging to class $$j$$ or actual output generated by the model. An input instance $${x}^{\left(i\right)}$$ belongs to the class equal to index of non-zero element in vector $${y}^{\left(i\right)}$$.

### Depthwise separable convolutions for 1-D signal

**Fig. 4:**
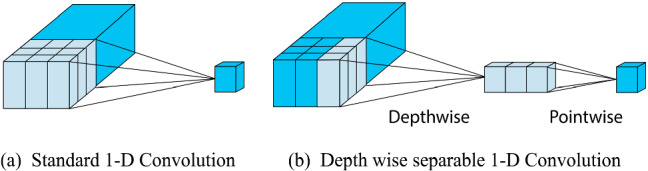
Operating principle illustration **a** Standard 1-D convolution layer, **b** Depthwise separable 1-D convolution layer

Depthwise separable convolution was developed by Sifre et al. to reduce the model size and increase the convergence speed of AlexNet in 2013 [34]. The widespread application of depthwise separable layer is Xception (Extreme Inception) architecture. The design of the depthwise separable layer is based on the hypothesis that inter-channel convolution and spatial convolutions are strictly independent of each other, and hence the operations can be separated. Therefore, in depthwise separable layers, first spatial convolution is performed on each channel followed by inter-channel convolution, also known as pointwise convolution. The difference between standard convolution and depthwise separable convolution is represented by Fig. 4.

Equations (4) and (7) are mathematical representations of standard Convolution layer Conv1D and depthwise separable Convolution layer SeparableConv1D*.* Here *k* is the kernel size, *c*_*i*_ and *c*_*o*_ are the numbers of input and output channels.

4 $$ \begin{aligned} y_{{i,c_{{_{0} }} }} & = {\text{Conv}}1{\text{D}}(W_{{k,c_{i} ,c_{0} }} ,x_{{i,c_{i} }} ) \hfill \\ &  = \sum\nolimits_{{K,C_{{}} }}^{K,F} {W_{{k,c_{i} ,c_{0} }} ,x_{{(i + k,c_{i} )}} } \hfill \\ \end{aligned} $$

5 $$ Np(C{\text{onv}}1{\text{D}}) = k \times Ci \times Co, $$

6 $$ No({\text{Conv}}1{\text{D}}) = l_{i} \times Np({\text{Conv}}1{\text{D}}), $$

7 $$ \begin{aligned} y_{{i,c_{o} }} & = {\text{SepConv}}1{\text{D}}(W_{{c_{o} }} ,W_{{k,c_{i} }} ,x_{{i,c_{i} }} ) \hfill \\ & = {\text{point wise }} {\text{convolution}}\left( {W_{{c_{o} }} ,{\text{depth wise }} {\text{convolution}}\left( {W_{{k,c_{i} }} ,x} \right)} \right) \hfill \\ & = \sum\nolimits_{{c_{i} }} {W_{{c_{o} }} \cdot \left( {\sum\nolimits_{k}^{k} {W_{{k,c_{i} }} \cdot } x_{{\left( {i + k,c_{i} } \right)}} } \right)} , \hfill \\ \end{aligned} $$

8 $$ Np(Sep{\text{Conv}}1{\text{D}}) = k \times Ci + Ci \times Co4, $$

9 $$ No(Sep{\text{Conv}}1{\text{D}}) = l_{i} \times Np(Sep{\text{Conv}}1{\text{D}}). $$

Equations (5) and (6) represents the number of parameters and operations required in the standard 1-D convolution layer. Similarly, Eqs. (8) and (9) represents the number of parameters and operations required in the 1-D depthwise separable convolution layer. Equation (10) represents the reduction in the depthwise separable layer’s network parameters compared to the standard convolution layer. It can be deduced that for the same number of output filters, the larger the receptive field, the lesser the number of parameters (*N*_*p*_) and the computational cost (*No*) model.

10 $$ \frac{{Np(SepC{\text{onv}}1{\text{D}})}}{{Np(C{\text{onv}}1{\text{D}})}} = \frac{k \times Ci + Ci \times Co}{{k \times Ci \times Co}} = \frac{Co + k}{{k \times Co}}. $$

### Spectral features

Wavelet transform has been a popular approach for the analysis of non-stationary signals. It has the advantage of localization of signal in time as well as frequency plane. We used a discrete wavelet transform (DWT) with mother wavelet ‘db6’. In DWT, the scale (a) and translational (b) parameters are discretized on a dyadic scale. The DWT can be calculated as

11 $$ S_{{2^{j} }} x(n) = \sum\nolimits_{k \in j} {h_{k} S_{{2^{j - 1} }} x(n - 2^{j - 1} k)} , $$

12 $$ W_{{2^{j} }} x(n) = \sum\nolimits_{k \in j} {g_{k} S_{{2^{j - 1} }} x(n - 2^{j - 1} k)} , $$

**Fig. 5:**
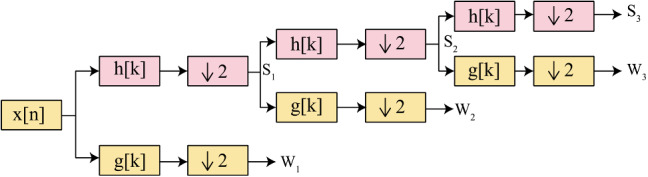
Multi-resolution wavelet decomposition

*S*_*2j*_ is smoothing operator, and *W*_*2j*_ is wavelet transform of discrete signal *x*[*n*]. Here *h*_*k*_ and *g*_*k*_ are coefficients of LPF and HPF, respectively. *S*_*2j*_ and *W*_*2j*_ are also known as approximation and detail coefficients, respectively. The wavelet functions are generally orthonormal. We have implemented the multi-resolution wavelet transformation given by Eq. (13) and illustrated by Fig. 5. Multi-resolution is a process in which signal can be decomposed on the next scale by using the approximation of signal on the previous scale [40]

13 $$ S_{{2^{j - 1} }} x(n) = \sum\nolimits_{k \in j} {h_{k} S_{{2^{j} }} x(n - 2^{j} k) + } \sum\nolimits_{k \in j} {g_{k} S_{{2^{j} }} x(n - 2^{j} k)} . $$

### Model optimization

The proposed model is optimized using the Sequential Model based Global Optimization (SMBO) algorithm [41]. We have used “Hyperopt”, a python library, to implement optimization algorithms based on SMBO. It offers the advantage of parallelization and handling hundreds of variables in a cost-effective manner [37]. In O-WCNN following parameters are optimized: number of nodes in the dense layer, number of dense layers, dropout probability, and batch-size. We have used “Hyperas” for defining the search space and searching for an optimized model. The “Hyperas” is a wrapper function used for fast prototyping of “hyperopt” with Keras models [42].

## Structure of proposed model

### O-WCNN architecture

**Fig. 6:**
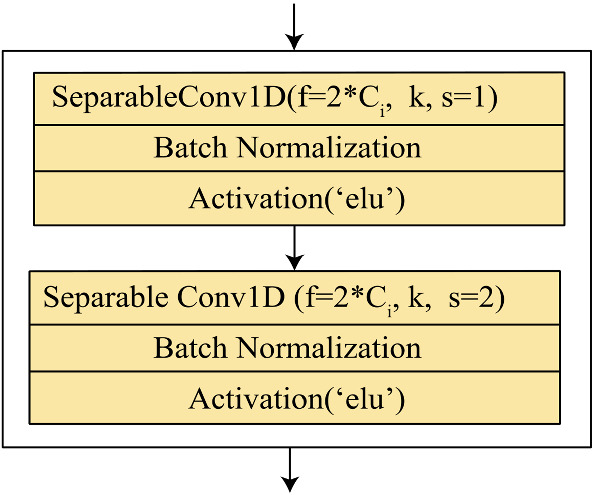
Functional unit of O-WCNN

**Fig. 7:**
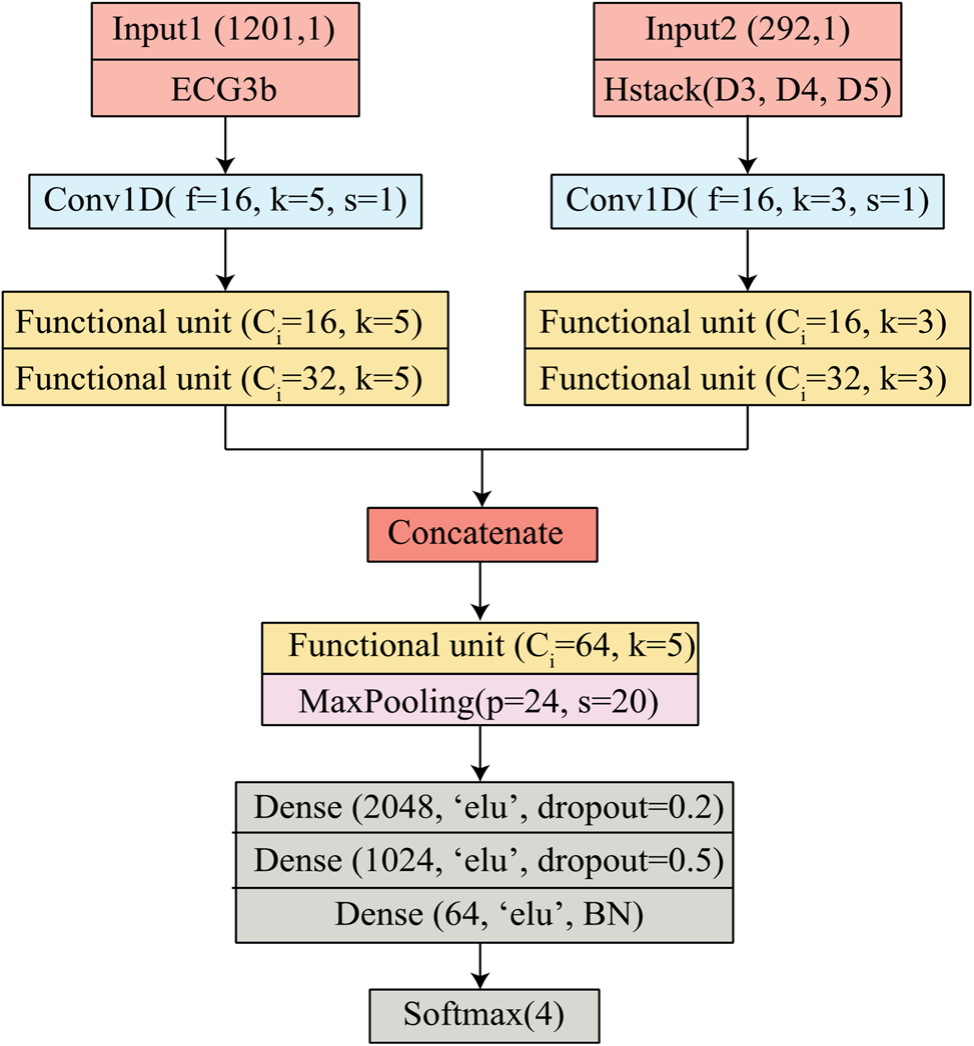
Proposed O-WCNN architecture

The proposed multi-channel model is designed such that it concatenates spectral and spatial features into a single model. Here one channel composed of functional unit extracts the spatial features, whereas the spectral information is added using the wavelet transformed coefficients as input on the other channel. The proposed O-WCNN model design is an adaption of mVGGNet architecture [36], replacing depth-wise separable convolution layers in place of conventional convolution layers. Figure 6 represents the functional unit of our model. It is made of two depthwise separable convolution layers followed by batch normalization and activation layers. The use of two convolution layers increases the receptive field of the model. The second separableConv1D is used with a stride value equal to two. It reduces feature map size by half at the output from the functional unit. The complete network model is shown in Fig. 7. The 3-beat ECG segment, as well as horizontally stacked detail coefficients D3–D5, are single-channel 1-D signals. Therefore, conventional Conv1D layers are used first to increase the depth of feature map. Using separate functional units for Input 1 and Input 2 allows multi-scale feature extraction.

For channel 1 subjected to Input1, kernel size is chosen as 5. For channel 2 subjected to Input 2 kernel size is chosen as 3. Here Input1 is 3-beat segmented ECG signal, and Input 2 is horizontally stacked coefficients D3-D5. The size of Input 1 and Input 2 is 1201 and 292, respectively. The kernel size of channel 2 is chosen as smaller compared to channel 1 due to the smaller size of Input 2. Each functional unit generates a feature map of depth two times the number of channels in the input data. The multi-scale spatial and spectral features are concatenated after two functional units.

The network comprises a total of twelve convolution layers (two conventional convolution layers and ten depthwise separable layers), ten activation and batch normalization layers, one MaxPooling layer followed by four Dense layers. Last layer is the softmax layer with a number of nodes equal to the number of target classes. The hyperparameters of fully connected layers are optimized using “Hyperopt” library [37].

The proposed architecture is different from mVGGNet [36] in terms of effective utilization of network parameters using depthwise separable convolution layers. The network can learn from both spectral and spatial features, resulting in improved performance of the model.

### Model parameter selection

*Activation Function*: the convolutional feature maps are processed by a non-linear activation function for faster convergence of the training algorithm. Therefore, we have used the Exponential Linear Unit ‘elu’ as an activation function represented by Eq. (14). This activation function is selected after experimenting with various activation functions in our prior work and concluded that using “elu” reduces the information loss contained in the biomedical signals’ negative peaks.

14 $$ f(x) = \left\{ \begin{gathered} x,\quad {\text{for}}(x > 0) \hfill \\ \alpha *(\exp (x) - 1,\quad for(x \le 0) \hfill \\ \end{gathered} \right\}. $$

*Optimization Algorithm*: the optimization algorithm used for compilation and model training is *Stochastic Gradient Descent (SGD)* [23, 43–45]. We have implemented a decaying learning rate schedule as given by Eq. (15). After every 30 epochs, the learning rate is decayed by a factor of 0.1. The initial learning rate ${\eta }_{0}$ is empirically set as 0.01. The batch size was set as 64 for relaxing the memory requirements during each epoch.

*Overfitting*: batch normalization and dropout layers are used in the architecture to avoid overfitting. Further, early stopping is implemented to evade overfitting by stopping the model training if validation accuracy stops improving for 20 epochs. The maximum number of epochs for training is set as 200. Dropout layers play a significant role in improving the generalization ability of the model by avoiding overfitting. We have used two dropout layers with dropout probability of 0.5 and 0.2. To further avoid over-fitting, Batch normalization is also used.

15 $$ \eta = \eta_{0} *0.1^{\frac{N}{30}} . $$

## Experiment and evaluation

### Dataset

The standard MIT-BIH database is used in this work to evaluate the model's performance [46]. As this database is available publicly since 1980, most of the methods in literature have been analyzed using this database. So, it is a popular choice to compare the proposed model performance with state-of-the-art models. This database is a collection of 48 two-channel digitized recordings sampled at 360 Hz. Each recording is 30 min long, having 648,000 samples. The database has approximately 110,000 heartbeats labeled into 15 types of arrhythmia conditions by experts. Out of 48 recordings, 23 recordings are randomly selected from 24-h ambulatory recordings. The remaining recordings are carefully selected to represent the clinically significant arrhythmias. Like other biomedical signal databases, this database also has an imbalanced distribution of normal and arrhythmic beat instances, creating biased results from the classifier.

### Evaluation scheme

The proposed method is evaluated using both subject-oriented inter-patient and class-oriented intra-patient evaluation scheme. For inter-patient evaluation, total 44 recordings from the MIT-BIH database (leaving the recordings with paced beats) are organized into two patient-independent datasets- Training set referred to as DS-I and Test set referred to as DS-II inspired by Chazel et al. [47]. The distribution of recordings into dataset DS-I and DS-II is given in Table 2. The recordings are divided into two sets in approximately 70:30 ratio. The bigger training set than [42] reduces overfitting and the need for an augmented dataset. The datasets are collection of labelled segmented heartbeats belonging to four arrhythmic classes grouped as per the American National Standard (ANSI/AAMI EC57:1998) [48]. Refer to Table 3 for grouping of 15 types of arrhythmia cases into four classes (except Q class) as per AAMI standard. Table 3 also provides the distribution of heartbeat examples in training set DS-I and test set DS-II.

**Table 2:** Distribution of recordings from dataset MIT-BIH into a training set DS-I and test set DS-II

DS-I	101, 103, 105, 106, 109, 111, 113, 115, 117, 119, 121, 122, 123, 200, 202, 203, 207, 210, 212, 213, 214, 219, 221, 222, 228, 230, 231, 232, 233, 234
DS-II	100, 108, 112, 114, 116, 118, 124, 201, 205, 208, 209, 215, 220, 223

**Table 3:** AAMI recommended grouping of 15 types of arrhythmia into 5 classes [35]

AAMI heartbeat class	Details	Number of heartbeats	
		DS-I	DS-II
Normal (N)	Normal beats (NOR), Left Bundle Branch Block Beats (LBBB), Right Bundle Branch Block Beats (RBBB), Atrial Escape Beats (AE), Nodal (junctional) Escape Beats (NE)	61,911	27,850
Ventricular Ectopic Beats (VEB)	Premature Ventricular Contraction (PVC), Ventricular Escape beats (VE)	5346	1659
Supraventricular Ectopic Beats (SVEB)	Atrial Premature Beats (AP), aberrated Atrial Premature beats (aAP), Nodal (junctional) Premature beats (NP), Supraventricular Premature beats (SP)	2214	807
Fusion Beats (F)	fusion of ventricular and normal beat	405	397
Total number of beats		69,876	30,713

The four classes (except Q-beat) and their example distribution into the training set DS-I and DS-II are given

For class-oriented evaluation, the datasets DS-I and DS-II instances are combined to make a single dataset DS having 100,589 ECG segments belonging to four classes. Stratified slices of dataset DS are used for training, validation, and testing. Generally, the class-oriented intra-patient evaluation protocol generates over-estimated results due to the presence of intra-patient heartbeats in the training set and test set.

This protocol is used here only for comparison with the recent similar works. The four target classes used are Normal beats (N), Supraventricular Ectopic beats (SVEB), Ventricular Ectopic beats (VEB), and fusion beats (F).

In this paper, we have limited the scope of analysis to only a single lead, modified limb lead (ML-II). Lead ML-II is preferred over lead V1 due to better projection of ventricle impulses in the QRS complex of lead ML-II.

### Data imbalance

Generally, models trained on the MIT-BIH database tend to bias toward majority class instances due to imbalanced data. The minority class examples are merely treated as outliers due to a huge imbalance between majority class and minority class instances [25]. Here, the arrhythmic classes are minority classes, whereas normal class examples are in the majority. Therefore, the model tends to mispredict an arrhythmic class example to a normal class. To reduce misclassification errors due to class imbalance, the model used scikit-learn's module class_weight. It assigns a higher weight to minority class inversely proportional to the number of examples of that class and ensures better learning for misclassified examples belonging to the minority class. The assigned class weights are [0.28 3.26 7.89 43.13].

### Cross-validation

In this work, the model is trained and tested with tenfold stratified cross-validation. The train set is partitioned into ten stratified subsets such that every subset has the same proportion of all types of arrhythmic beats. For *k*th validation, (*k*–1) subsets are used for training, and the remaining exclusive subset is used for validation and early stopping. The process is repeated k times, choosing a different validation set each time. The reported model performance was evaluated on the test set DS-II after each validation.

### Performance metrics

The following statistical measures are given by Eqs. (16–21) are used for performance analysis of the proposed O-WCNN model.

16 $$ {\text{Sensitivity}} = \frac{{{\text{TP}}}}{{{\text{TP}} + {\text{FN}}}}, $$

17 $$ {\text{Specificity}} = \frac{{{\text{TN}}}}{{{\text{TN}} + {\text{FN}}}}, $$

18 $$ \Pr {\text{eision}}({\text{ppv}}) = \frac{{{\text{TP}}}}{{{\text{TP}} + {\text{FP}}}}, $$

19 $$ {\text{Accuracy}}({\text{acc}}) = \frac{{{\text{TP}} + {\text{TN}}}}{{{\text{TP}} + {\text{FP}} + {\text{TN}} + {\text{FN}}}}, $$

20 $$ F1{\text{-Score}} = (2*{\text{TP}})/(2*{\text{TP}} + {\text{FP}} + {\text{FN}}), $$

21 $$ G{\text{-Score}} = \sqrt {{\text{Sensitivity}}*{\text{Specificity}}} . $$

Here, TP, FP, TN, and FN stand for True Positive, False Positive, True Negative, and False Negative, respectively.

### Experimental environment

The model is designed using Keras version 2.2.4. It is an open-source deep-learning library designed on TensorFlow framework. The data preparation step is completed using MATLAB R2016a. Data preparation, model design, training, testing, and final performance evaluation has been done on a single machine. The system configurations are given in Table 4.

**Table 4:** System configuration

Sr. no.	System Parameter	System Configuration
1	Processor	Intel(R) Core (TM) -i7 8750H 8th Gen
2	Speed	2.20 GHz
3	RAM	8 GB
4	GPU	1 × NVIDIA GeForce GTX 1050 Ti with 768 CUDA cores and 4 GB standard memory configuration
5	Operating System	Window 10

## Results and discussion

The proposed model is evaluated using two approaches. (1) Subject-oriented inter-patient approach and (2) class-oriented intra-patient approach. In the inter-patient approach, the complete dataset was divided into two datasets DS-I and DS-II, such that both datasets have recordings belonging to separate patients as proposed in [42]. However, in the intra-patient approach, the data set DS1 and DS2 are combined into a single dataset DS, confirming that training and testing sets may have signals belonging to the same patients.

### Subject-oriented inter-patient evaluation

The model is evaluated with tenfold cross-validation. The dataset DS-I is used for training and validation. However, the model performance is tested on the test set DS-II after each cross-validation. The average test set results are reported in this paper.

The confusion matrix and related performance measures are given in Table 5. The model average prediction accuracy is 99.43%. Whereas the overall F-Score is 98.86%. The model performance is compared with other state-of-the-art methods based on CNN. These models are compared based on efficacy in generalizing the examples belonging to VEB and SVEB classes. The ECG arrhythmia classification is challenging due to variation in inter-beat and intra-beat morphologies belonging to different patients. Therefore, inter-patient arrhythmia classification models are compared based on how better the model generalizes on arrhythmic classes, especially VEB and SVEB classes. The comparative results are represented in Table 6. The results of other methods are the same as they are reported in the literature. However, some methods VGGNet [49], MS-CNN [23] are implemented, and results are compared in Table 7. The models are also compared based on F1-Score and G-Score measure given in Table 8. It can be deduced from Tables 6, 7 and 8 that the O-WCNN model outperforms the previously proposed model mVGGNet [36] and other state-of-the-art methods. Its enhanced performance is due to the inclusion of spectral features and efficient utilization of model parameters in depthwise separable convolution layers. Further performance enhancement is due to hyperparameter optimization, such as the number of nodes in fully connected layers, the number of fully connected layers, dropout probability, batch size, and learning rate.

**Table 5:** Confusion matrix and performance measures for the model tested using subject-oriented inter-patient evaluation approach

	Predicted class					Accuracy (%)	Sensitivity (%)	Specificity (%)	Precision (%)	F1-Score (%)
		N	V	S	F					
Actual class	N	27,721	56	68	5	99.17	99.54	99.54	99.55	99.55
	V	47	1592	13	7	99.48	95.96	99.68	94.54	95.24
	S	34	11	750	12	99.46	92.94	99.63	87.31	90.04
	F	43	25	28	301	99.61	75.82	99.92	92.62	83.38

**Table 6:** Performance comparison of proposed architecture with existing methods using the subject-oriented inter-patient approach

Reference	VEB				SVEB			
	Accuracy (%)	Sensitivity (%)	Specificity (%)	Precision (%)	Accuracy (%)	Sensitivity (%)	Specificity (%)	Precision (%)
Kiranyaz et al. [20]	98.6	95	98.1	89.5	96.4	64.6	98.1	62.1
Xia et al. [27]	94.9	83.3	93.9	57	94.4	17.6	93.7	51.6
Xia et al. [22]	95.7	59.3	94.9	64.3	95.8	86.1	94.5	84.3
Jangra et al. [36]	98.79	91.6	99.29	89.92	99.16	82.81	99.62	84.3
Chen et al. [51]	99.09	88.84	**99.80**	**96.78**	97.92	63.90	99.23	76.10
Romdhane et al. [50]	99.35	94.54	99.70	95.73	99.15	77.88	**99.71**	**87.65**
Proposed Method	**99.48**	**95.96**	99.68	94.54	**99.46**	**92.94**	99.63	87.31

Bold values represent the maximum value of that column

**Table 7:** Performance comparison of proposed architecture with existing methods using inter-patient evaluation approach

Reference	VEB				SVEB			
	Accuracy (%)	Sensitivity (%)	Specificity (%)	Precision (%)	Accuracy (%)	Sensitivity (%)	Specificity (%)	Precision (%)
VGGNet [49]	97.39	74.2	99.03	84.46	97.23	33.35	98.2	53.57
MSCNN [23]	98.02	79.69	98.7	89.19	97.53	53.88	99.62	64.26
mVGGNet [36]	98.79	91.6	99.29	89.92	99.16	82.81	99.62	84.3
Proposed method	99.48	95.96	99.68	94.54	99.46	92.94	99.63	87.31

The methods are implemented on the same machine for a fair comparison

**Table 8:** Performance comparison based on F-Score and G-Score measures using inter-patient evaluation approach

Reference	VEB		SVEB	
	F–Score (%)	G-Score (%)	F-Score (%)	G-Score (%)
Kiranyaz et al. [20]	92.2	96.5	63.3	79.6
Xia et al. [27]	67.7	88.4	26.2	40.6
Xia et al. [22]	61.7	75.0	85.2	90.2
Jangra et al. [36]	90.8	95.4	83.5	90.8
Chen et al. [51]	92.6	94.2	69.5	79.6
Romdhane et al. [50]	95.1	97.1	82.5	88.1
VGGNet [49]	79.0	85.7	41.1	57.2
MSCNN [23]	84.2	88.7	58.6	73.3
mVGGNet [36]	90.8	95.4	83.5	90.8
Proposed Method	95.2	97.8	90.0	96.2

The computational complexity of O-WCNN is greater than mVGGNet due to the imposition of spectral features in the model. The increase in computational complexity is justified by enhancement in the performance of the proposed model. The proposed model is still computationally efficient with the number of parameters, only 30.8% of the number of parameters in VGGNet and MSCNN.

**Fig. 8:**
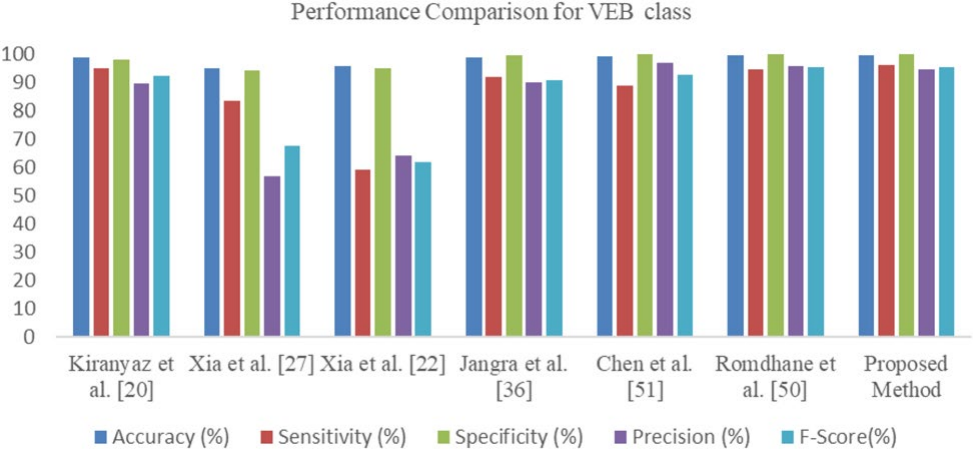
Performance comparison representation for classification of VEB vs Non-VEB class

**Fig. 9:**
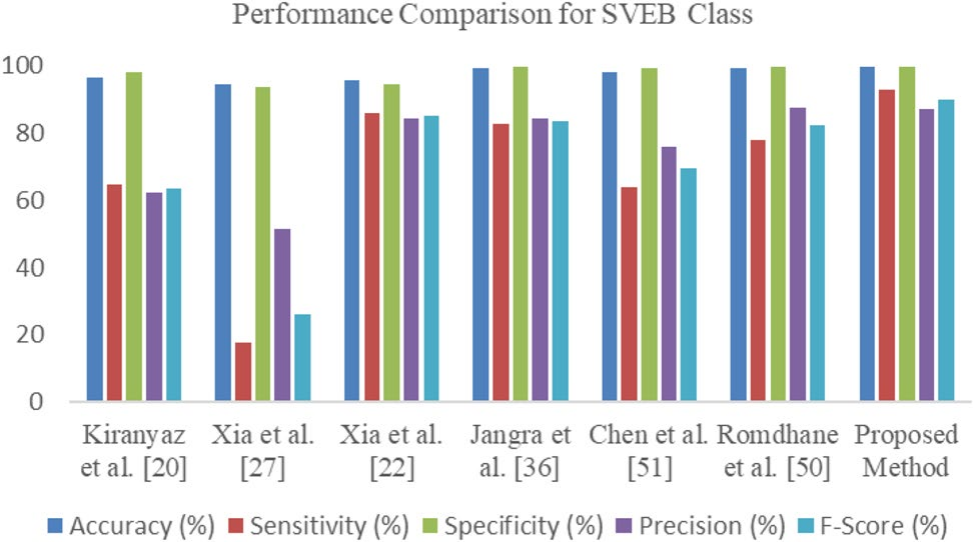
Performance comparison representation for classification of SVEB vs Non-SVEB class

It should be noted that the accuracy and sensitivity of the model are more significant than the other state-of-the-art models. The O-WCNN model correctly classifies with 99.48%, 99.46% accuracy for VEB and SVEB classification, respectively. The model has 95.96% and 92.94% sensitivity for VEB and SVEB classification, respectively. The values of specificity and precision are 99.68% and 94.54% for VEB class. The specificity and precision values are 99.63% and 87.31% for SVEB classification. The same results are plotted using a bar chart in Figs. 8 and 9 for visual interpretation of results. The improvement in the performance of O-WCNN is contributed by the use of spectral features, depthwise separable convolution layers, and optimization of hyperparameters using 'Hyperas', a wrapper for 'Hyperopt' library [48].

The model is also evaluated using class-oriented evaluation for comparison with the similar methods reported in the literature. The complete dataset DS is used here for training, validation, and testing. 80% of data is used for training and validation using tenfold cross-validation, and 20% stratified data is used for testing. Due to shuffling of the dataset DS, the training and test set may have ECG beats belonging to the same patients. The comparative results of the class-oriented evaluation scheme are given in Table 9. The results are the average of results obtained after each cross-validation. It can be seen that the proposed model outperforms all other similar models. The model proposed by Bouny et al. W-MSCNN is nearest to the proposed model O-WCNN. But our method has shown higher average sensitivity as compared to W-MSCNN.

**Table 9:** Performance comparison of proposed architecture with existing methods using class-oriented evaluation approach

Reference	Method	Accuracy (%)	Average sensitivity (%)	Average precision (%)	Average F1-Score (%)
Martis et al. [9]	DWT + SVM	93.8	91.5	87.9	89.06
Bouny L. et al. [24]	MS-WCNN	99.11	93.54	96.72	–
Mahmud et al. [25]	1D-CNN (DeepArrNet)	99.28	99.13	99.08	99.11
Chen et al. [51]	MF-CBRNN	99.56	95.90	97.14	96.40
Qiao et al. [52]	DELM-LRF-BLSTM	99.32	97.15	98.30	97.71
Xu and Liu [53]	1-D CNN	99.43	94.30	97.99	96.03
Proposed	O-WCNN	99.58	99.2	99.15	99.28

W-MSCNN uses SWT decomposed coefficients with mother wavelet ‘db1’. SWT tends to generate redundant coefficients. The choice of mother wavelet and redundant coefficients may have hampered the sensitivity of the model. We have implemented DWT-based decomposition using mother wavelet ‘db6’, which is most suited for ECG-based applications due to a morphological similarity with the mother wavelet. Mahmud et al. proposed DeepArrNet using depthwise separable convolution layers. Our model O-WCNN has shown comparable performance to DeepArrNet without using an augmented dataset.

**Fig. 10:**
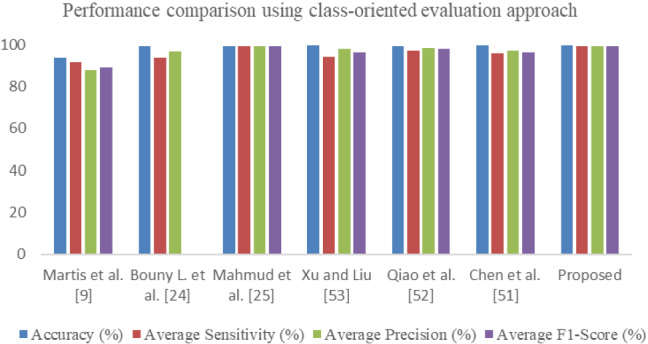
Performance comparison using class-oriented evaluation approach

**Fig. 11:**
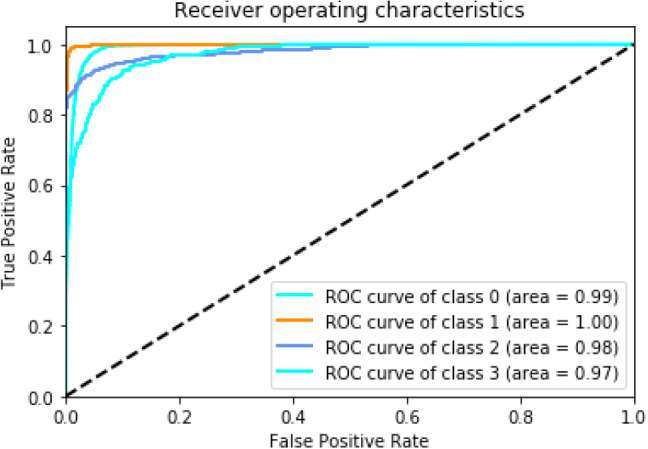
Receiver operating characteristic curve of the proposed method using class-oriented evaluation

It is clear from Table 9 that O-WCNN outperformed the existing methods using a class-oriented evaluation protocol. The increment is due to spectral and spatial feature maps learning by using wavelet decomposed detail coefficients, optimization of hyper-parameters, and better utilization of network parameters by using depthwise separable layers. This point should be highlighted that the class-oriented evaluation has generated better results than inter-class evaluation as expected due to the presence of signals in training and testing sets belonging to the same patient. However, this evaluation protocol has some practical constraints. There is a very high probability that the subject recommended for diagnosis has completely different morphology from the data on which the model is trained. However, the model can be designed using patient-specific evaluation and used in remote and portable mobile units for ECG analysis. Figure 10 is a bar chart representation of Table 9. Figure 11 represents the receiver operating characteristic (ROC). Area under the curve (AUC) is 0.99, 1.0, 0.98 and 0.97 for class N (class 0), VEB (class 1), SVEB (class 2) and F (class 3) respectively. The value of AUC closer to 1 is an indicator of good performance of the proposed O-WCNN model.

## Conclusion

In this paper, an effective deep convolutional neural network is proposed for cardiac arrhythmia classification. The proposed multi-channel model can concatenate the spatial feature map and spectral feature map. The ECG signal is pre-processed using wavelet-based denoising. The clean segmented ECG signal was used as Input1 for one channel. The wavelet transformed coefficients (D3, D4, and D5) extracted from clean segmented ECG were horizontally stacked and given as Input2 on the second channel. The network was designed using depthwise separable 1-D convolution layers. Further, the hyperparameters of the model are optimized using the SMBO algorithm. The proposed model O-WCNN is evaluated using both the subject-oriented inter-patient and class-oriented intra-patient evaluation approach. The model is trained and validated using tenfold cross-validation. Further to reduce overfitting, the model is equipped with dropout layers, batch normalization layers, and early stopping in the training schedule. The proposed O-WCNN achieved 99.48% and 99.46% accuracy for ventricular ectopic beats (VEB) and supraventricular ectopic beats (SVEB) classification using inter-patient evaluation. However, the model reported enhanced performance with a class-oriented intra-class evaluation approach. The work concludes that the optimized integration of spectral and spatial features through an optimized 1-D convolutional neural network can significantly enhance an ECG arrhythmia classifier’s performance.

## Data Availability

The code is available upon request.
